# Clinical and instrumental assessment of the effects of a new product based on hydroxypropyl chitosan and potassium azeloyl diglycinate in the management of rosacea

**DOI:** 10.1111/j.1473-2165.2011.00598.x

**Published:** 2012-03

**Authors:** Enzo Berardesca, Matilde Iorizzo, Elva Abril, Giancarlo Guglielmini, Maurizio Caserini, Renata Palmieri, Gérald E Piérard

**Affiliations:** 1 San Gallicano Dermatological InstituteRome, Italy; 2 Private Practice, DermatologyLugano, Switzerland; 3 Scientific/Regulatory Affairs DepartmentSinerga S.p.A., Milan, Italy; 4 Scientific DepartmentPolichem SA, Lugano, Switzerland; 5 Department of Dermatopathology, University Hospital of LiègeLiège, Belgium

**Keywords:** Azeloglicina®, erythema, potassium azeloyl diglycinate (PAD), rosacea, skin hydration

## Abstract

**Summary:**

**Background:**

Rosacea is a chronic inflammatory skin disease affecting mostly facial skin. Its origin is multifactorial. Important steps in its treatment are avoidance of any triggering factor and control of skin inflammation.

**Aim:**

To assess the benefit of topical applications of a new product (P-3075).

**Patients/Methods:**

A randomized, multicenter, double-blind, placebo-controlled, parallel-group, pilot study was carried out to evaluate the efficacy and tolerability of a cream (P-3075) based on 5% potassium azeloyl diglycinate (PAD, Azeloglicina®) and 1% hydroxypropyl chitosan (HPCH). Forty-two patients (rosacea stages I and II) were enrolled and randomized, 28 in the P-3075 group and 14 in the placebo group. They were asked to apply the cream twice daily for 4 weeks. The main assessments were the objective quantification of erythema and skin hydration using the Mexameter® and Corneometer® devices, respectively. Clinical signs and symptoms were evaluated on a four-point scale.

**Results:**

The P-3075 cream applied for 28 days was effective in skin protection by reducing erythema, evaluated both instrumentally and clinically. In addition, the clinical assessments of other symptoms such as flushing, stinging, and burning supported the beneficial effect of the P-3075 cream.

**Conclusions:**

The anti-inflammatory and moisturizing effects of potassium azeloyl diglycinate combined with the protective properties of HPCH allow the new product to be a good candidate for controlling signs and symptoms of rosacea.

## Introduction

Rosacea is a common chronic inflammatory skin disease that predominantly affects the face of fair-skinned phototype I and II Caucasians from northern European origin. Up to 1/3 report a family history of the disorder.^1^ Furthermore, it was reported to affect over 10 million Americans. In addition, the disease commonly affects southern Europeans, Asians, and African-Americans.^2^ Rosacea is quite common in middle-aged people, although it develops at younger age.

The skin condition is characterized by flushing, persistent erythema, inflammatory papules, pustules, nodules, edema, and telangiectasia. Associated features frequently include burning and stinging sensation on the face.^3^ The pathomechanism of rosacea is poorly understood, and there are no specific biologic markers for the disease. Recent molecular studies in patients with rosacea suggested alterations in the innate immune response. Vascular hypereactivity appears to be the primary phenomenon, exacerbated by inflammation. Fibroblast growth factor (FGF) and vascular endothelium growth factor (VEGF) are the main angiogenic mediators involved in this disorder.^4^ Ultraviolet (UV) light is the main agent stimulating proinflammatory cytokines, growth factors, and angiokines.^5^, ^6^ In addition, endocrine, psychological, pharmacologic, immunologic, infectious, thermal, and alimentary factors contribute to produce some vascular instability and tissue damage.^7^

Clinically, rosacea is classified in four subtypes based on the predominant clinical features, ranging from facial erythema (Stage I/II) to obvious inflammatory lesions (Stage III/IV).^3^, ^8^ Symptoms of rosacea include skin dryness and sensations of skin sensitivity, stinging and burning. An important step in rosacea management is avoidance of any potential triggering factor.^8^

Potassium azeloyl diglycinate, obtained by reacting the chloride of azelaic acid with two molecules of glycine and KOH, is a new generation ingredient. It is an innovative water-soluble molecule exhibiting sebum normalizing activity and whitening properties.^9^ The glycine content provides moisturizing effects and increases the stratum corneum plasticity. Hydroxypropyl chitosan (HPCH) is a water-soluble semisynthetic derivative of chitosan, which acts as a film protector for the skin from some environmental chemical or physical insults.^10^ HPCH is permeable to air and moisture, forming an elastic film and it increases the dispersion of other agents with a good safety profile. Chitosan and its derivatives possess adhesive properties on different biologic tissues because of their positive charges.^11^

## Materials and methods

During April and June 2009, 42 patients of both genders, aged 18–60 years and suffering from stage I and II rosacea, were enrolled after providing written informed consent. The study was a randomized, multicenter, double-blind, placebo controlled, parallel-group, pilot trial carried out to evaluate the efficacy and tolerability of the P-3075 cream (Polichem SA, Lugano, Switzerland) containing 5% potassium azeloyl diglycinate (Azeloglicina®;Sinerga S.p.A., Milan, Italy) and 1% HPCH.

Patients meeting the inclusion criteria were randomly allocated to P-3075 cream or placebo, corresponding to the moisturizing vehicle of the P-3075 cream. A 2:1 ratio in the respective numbers of panelists was considered to minimize the number of untreated patients. Patients were treated twice daily for 28 days (D) with a 2-week follow-up period. The assessments were performed at inclusion (baseline), at D7, D14, D28 (end of treatment), and D42 (end of follow-up).

At each visit, instrumental evaluations of erythema and stratum corneum hydration were performed on the forehead, cheeks, and chin by assessing the erythema index (Mexameter®; C+K electronic, Cologne, Germany) and skin capacitance (Corneometer® CM 825; C+K electronic), respectively. Both the Mexameter® and Corneometer® are noninvasive devices widely used for assessing skin conditions and the effects of topically applied products.^12^–^14^ Moreover, flushing, erythema, edema, itching, burning and stinging were evaluated on a four-point scale, where 0 = none, 1 = mild, 2 = moderate and 3 = severe.

Data were processed by means of the program sas® for Windows, Release 9.2 (SAS® Institute Inc., Cary, NC, USA). Descriptive statistics were performed including mean, standard deviation, percentage, coefficient of variation, minimum value, median and maximum value for continuous variables and frequencies, and percentages for categorical variables. Superior efficacy versus placebo was tested by the analysis of covariance (ancova). Categorical variables (signs and symptoms) were analyzed by means of chi-square test and Fisher Exact Test. *P* values < 0.05 were considered statistically significant.

## Results

Forty-two patients were randomized: 28 in the P-3075 group and 14 in the placebo group. Both groups were similar with respect to age and gender proportion (Table 1). None of the patients discontinued the trial and no major protocol violation was reported.

**Table 1:** Demographic characteristics

	P-3075	Placebo

Gender, N (%)		
Males	8 (28.6)	3 (21.4)
Females	20 (71.4)	11 (78.6)
Age, years		
Mean ± SD	40.3 ± 11.3	39.3 ± 11.3
Median	40	34
Range	20–60	26–60

**Figure 1:**
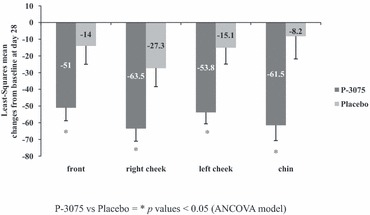
Evaluation of erythema by means of Mexameter pre–posttreatment. Least-Squares mean changes from baseline.

The mean erythema index in the ancova model showed a statistically significant reduction at the end of treatment in favor of P-3075 in all evaluated facial areas (Fig. 1). A significant decrease in erythema was present from D14 on, the difference versus baseline means were as follows: forehead −27.9 (*P* = 0.017), right cheek −24.2 (*P* = 0.042), left cheek −34.3 (*P* = 0.002), and chin −26.7 (*P* = 0.027). At D28, the difference versus baseline means were as follows: forehead −37 (*P* = 0.009), right cheek −36.1 (*P* = 0.010), left cheek −38.8 (*P* = 0.002), and chin −53.3 (*P* = 0.002).

Moreover, the composite erythema index representing the sum of the 4 site-specific erythema indices showed a statistically significant decrease at any time point in favor of the P-3075 formulation: D7 (−39.5, *P* = 0.016), D14 (113.0, *P* = 0.000), D28 (167.0, *P* = 0.000), and D42 (99.7, *P* = 0.030).

**Figure 2:**
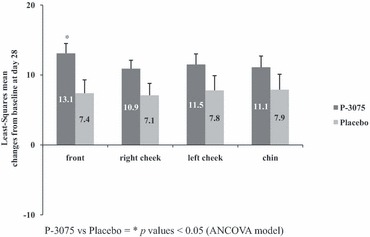
Evaluation of hydration by means of Corneometer pre–posttreatment. Least-Squares mean changes from baseline.

As expected, the mean stratum corneum hydration was consistently increased, on all facial sites both in the placebo and in P-3075 groups. Furthermore, the ANCOVA model showed a statistically significant increase in the P-3075 group from baseline to D14 (4.9, *P* = 0.030) and D28 (5.7, *P* = 0.020) on the forehead (Fig. 2).

An improvement was observed for all the signs and symptoms of rosacea in the P-3075 group from D7 to D42, compared to no changes in the placebo group.

**Figure 3:**
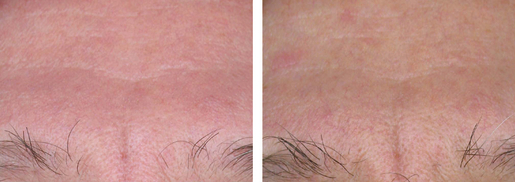
Front erythema improvement after 28 days of treatment with P-3075.

**Figure 4:**
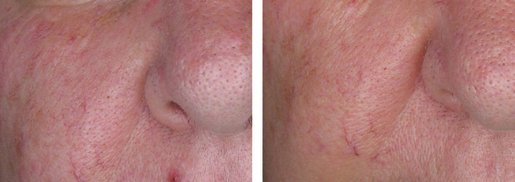
Right cheek erythema improvement after 28 days of treatment with P-3075.

A trend to erythema fading was observed in the P-3075 group from D7 to D42. In the P-3075 group, the clinical assessment of erythema showed a statistically significant decrease at D14 (*P* = 0.007 in the chi-square test and *P* = 0.005 in the Fisher exact test), at D28 (*P* = 0.005 in the chi-square test and *P* = 0.011 in the Fisher exact test), and at D42 (*P* = 0.001 in both the chi-square test and the Fisher exact test). Individual cases referred to the end of treatment are documented in Figures 3 and 4.

Moreover, a statistically significant decrease was detected at D7 for burning (*P* = 0.004 in the chi-square test and *P* = 0.009 in the Fisher exact test), at D14 for stinging (*P* = 0.014 in the chi-square test and *P* = 0.008 in the Fisher exact test), and flushing (*P* = 0.011 in both the chi-square test and the Fisher exact test).

No local adverse reaction was reported in both treatment groups. Compliance was excellent in all patients.

## Discussion

The results showed that P-3075 applied for 28 days in patients with stage I and II rosacea was effective in skin protection by reducing erythema, evaluated both instrumentally and clinically. Using the Mexameter, the superiority of P-3075 over placebo was reported at all facial sites starting from D14 until the end of treatment. Calculating the composite erythemal index revealed that a protective effect was present at D7 persisting until the end of follow-up.

Consistent with the instrumental evaluation, the clinical inspection confirmed the improvement of facial erythema from D14 to D42, disappearing in 95% of patients.

The clinical assessments of other symptoms such as flushing, stinging and burning confirmed the protective effect of the P-3075 formulation. The improvement of erythema, in most cases accompanied by flushing, burning, and stinging sensation, is considered crucial in patients with rosacea because most of them are concerned about its development and persistence.^3^

Further to the effect on erythema, the P-3075 formulation showed an improved stratum corneum hydration effect on all facial sites. This effect was because of the moisturizing base cream of P-3075, and it was not unexpectedly similar to that of the placebo group. Because approximately 12% of patients with rosacea experienced scaling, skin dryness, or rash while using topical azelaic acid and may show skin barrier disruption and sensorial irritation to topical products,^15^ the moisturizing effect of the P 3075 formulation appears a valid alternative to relieve some rosacea signs and symptoms.

As no local adverse events were neither reported by the patients nor noticed by investigators, the P-3075 formulation exhibited an excellent profile in terms of local tolerance.

In conclusion, the anti-inflammatory and moisturizing effects of potassium azeloyl diglycinate combined with the protective properties of HPCH allow this new product to be an advance in improving symptoms of rosacea.
